# The impact of integrated nutrition-sensitive interventions on nutrition and health of children and women in rural Tanzania: study protocol for a cluster-randomized controlled trial

**DOI:** 10.1186/s40795-018-0238-7

**Published:** 2018-09-06

**Authors:** Dominic Mosha, Chelsey R. Canavan, Alexandra L. Bellows, Mia M. Blakstad, Ramadhani Abdallah Noor, Honorati Masanja, Joyce Kinabo, Wafaie Fawzi

**Affiliations:** 1 Ifakara Health Institute, Dar es Salaam, Tanzania; 2 Africa Academy for Public Health, Dar es Salaam, Tanzania; 3 Department of Global Health and Population, Harvard T.H. Chan School of Public Health, Boston, MA USA; 4 Sokoini University of Agriculture, Morogoro, Tanzania; 5 Department of Nutrition, Harvard T.H. Chan School of Public Health, Boston, MA USA

**Keywords:** Agriculture, Health, Nutrition, Dietary diversity, Children, Women, Extension workers

## Abstract

**Background:**

Nutrition-sensitive interventions such as homestead production of diverse, nutrient-rich foods, coupled with behavior change communication, may have positive effects on the nutritional status and health of rural households engaged in agriculture, particularly among women and young children. Engagement of agriculture and health extension workers in these communities may be an effective way of delivering nutrition-sensitive interventions given the dearth of trained health care providers in many developing countries. This study aims to assess the effects of integrated homestead food production, food consumption and women’s empowerment interventions using a multi-sectoral approach on women’s and child’s health and nutrition.

**Methods:**

This is a cluster-randomized community-based prospective study set in Rufiji district, a rural area in Eastern Tanzania. Ten randomly selected villages within the Rufiji Health and Demographic Surveillance Site (HDSS) in Eastern-Tanzania were paired and randomly assigned to the intervention or control arm. The Rufiji HDSS dataset was used to randomly sample households with women of reproductive age and children 6–36 months. The intervention includes provision of small agricultural inputs, garden training support, and nutrition and health counseling. This is delivered by community health workers and agriculture extension workers through home visits and farmer field schools. There are three time points for data collection: baseline, midline, and endline. Primary outcomes are women’s and children’s dietary diversity, maternal and child anemia status and growth (child stunting, child wasting, women’s BMI, and women and child hemoglobin).

**Discussion:**

This integrated agriculture and nutrition intervention among rural farming households is a simple and innovative solution that may contribute to the reduction of undernutrition and disease burden among women and children in developing countries. Engaging already existing workforce in the community may facilitate sustainability of the intervention package.

**Trial registration:**

ClinicalTrials.gov NCT03311698, Retrospectively registered on October 17, 2017.

## Background

Maternal and child undernutrition remains one of the most challenging health problems in low and middle-income countries (LMICs) [1]. Together they account for more than 10% of the global burden of disease. Nearly half of all child deaths globally, equivalent to 3,000,000 lives lost annually, are attributable to malnutrition. While some improvements in nutritional status have been observed in recent years in many developing countries, including Tanzania, stunting remains a leading form of undernutrition [2]. Undernutrition early in life has devastating consequences to individual health and community development including increased risk of infections, impaired cognitive ability, and reduced school and work performance [3].

Inadequate dietary intake and poor weaning foods in young children, both of which are important causes of undernutrition, are still major problems in many LMICs. Daily meals often lack the diversity needed for supplying essential nutrients for normal growth and prevention of diseases [4, 5]. Other contributors to undernutrition such as diarrhea and recurrent pneumonia are highly prevalent. Suboptimal access to safe water, poor sanitation, and poor health care services also contributes to malnutrition and illness in many rural settings [6]. Thus, it is vital to consider holistic interventions to address maternal and child undernutrition in LMICs for effective and maximum impact in health and economic development.

### Importance of nutrition in women and children

Pregnant and lactating women are among the most vulnerable groups to suffer from undernutrition and micronutrient deficiencies because they have physiologically higher nutrient requirements that are often not met [7]. Women should have a sufficient diet, in terms of both quality and quantity, before and after conception to maintain replete nutrient stores that can sustain themselves and their children. Adequate diet during the preconception period and pregnancy reduces the risk of adverse maternal and newborn outcomes such as low birthweight, stillbirth, preterm, and impaired cognitive and motor development [8, 9].

Similarly, infants and young children have high nutrient requirements due to rapid growth and development [7]. During illness, they experience a heightened demand of nutrients to maintain optimally functional immune systems [10]. This is of particular importance in sub-Saharan Africa due to the high prevalence of infectious diseases, including malaria, HIV and respiratory infections among children under 5 years of age [11].

### Rationale for advancing homestead food production to improve nutrition and health

Investment in the agriculture sector in various countries in Africa has resulted in large increases in staple crops that are typically rich in carbohydrates but poor in micronutrient content [12]. Promoting the agricultural sector with a view of nutrition-sensitive productivity will assist in accelerating nutritional gains in the community, and enhance production of diverse crops that enrich the quality of diets including micronutrient-rich foods [13]. Pathways by which agriculture can influence nutrition include increased home production and household availability of nutrient-rich crops, increased dietary diversification, and increased income and access to food in markets. Financial gains at the household level from agricultural production may have important implications for women who have primary responsibility for food production in many LMICs [14]. This may have direct effects on women’s empowerment through improved access to resources, which may influence their ability to make informed decisions regarding health care and meeting essential needs for their children.

In rural settings, where most households are engaged in smallholder farming, homestead food production (HFP) interventions offer one of the most promising pathways towards improving livelihoods, the diversity of agricultural outputs, food security, and maternal and child nutrition. Small-scale, nutrition-sensitive agricultural interventions equip smallholder farmers with the tools and skills to cultivate home gardens and raise livestock. Home gardens promote production of nutrient-rich fruits and vegetables that can grow well in local conditions. Household members, particularly women, are trained in cultivating the crops and raising livestock [14]. HFP interventions have shown promising increases in food production and dietary diversity [15]. This can improve women and child nutrition directly through consumption, and also indirectly by raising household income, purchasing power for health services, availability of food in markets, and empowerment of women in society.

For maximum health and nutrition impact, HFP interventions should be coupled with behavior change communication campaigns that include nutrition and public health messages, especially among women. This will effectively improve knowledge on the importance of a healthy diet, dietary diversity, and best practices for food preparation, child feeding, and health seeking behavior [16]. However, little is known on the most effective nutrition-sensitive intervention approaches. Furthermore, there is a lack of clarity on which approaches are most effective and feasible for integrating nutrition in agriculture sectors, including within extension services [17].

This study evaluates the effects of integrated nutrition-sensitive interventions on the health and nutrition of children and women in rural Tanzania while exploring the incorporation of existing cadres of agricultural extension workers together with community health workers as a sustainable workforce for providing basic health and nutrition education in rural communities.

## Methods/design

### Study setting

**Fig. 1:**
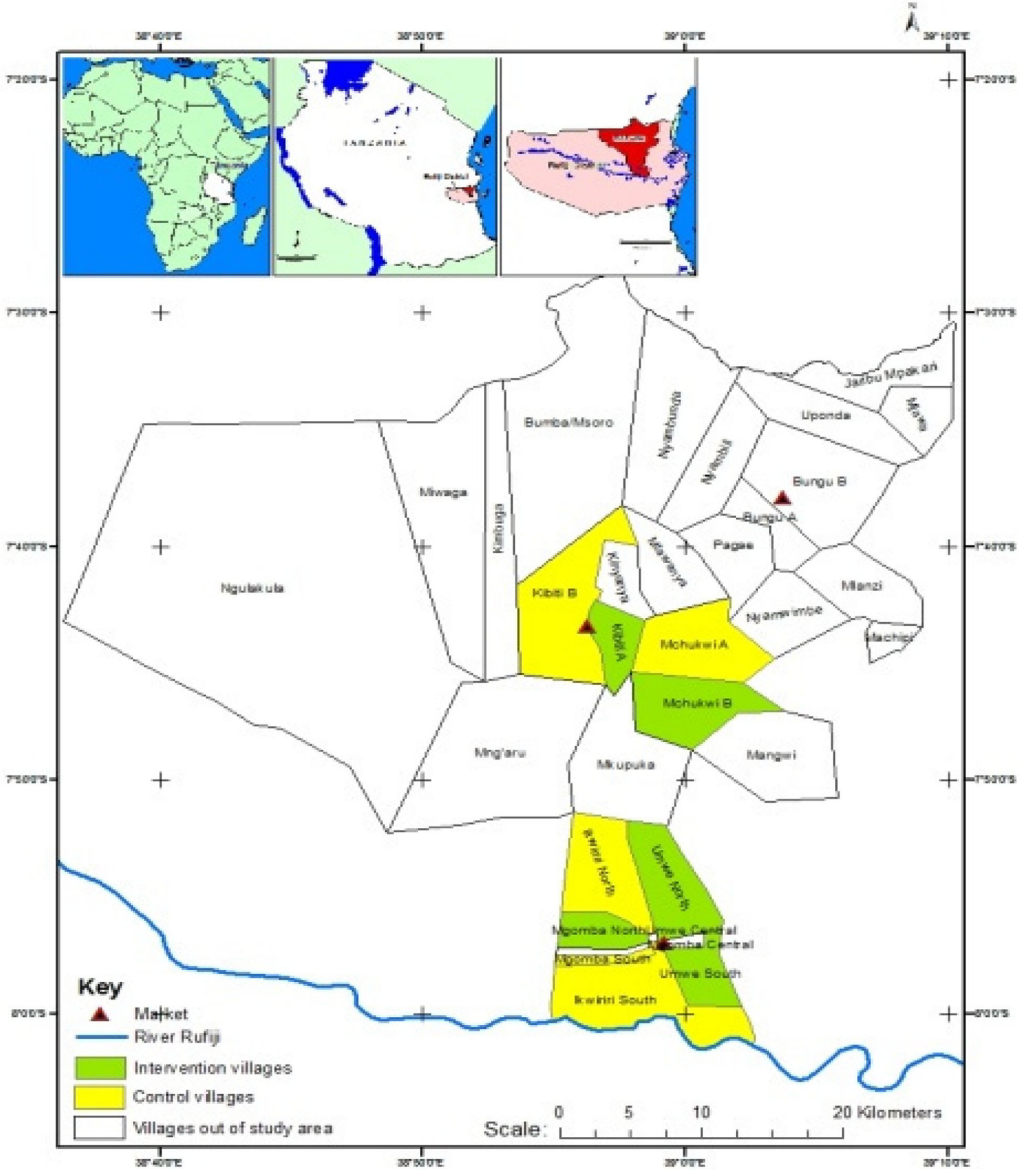
Map of the study area in Rufiji, Tanzania

The study is implemented in Rufiji district, Tanzania. Rufiji is one of six districts in the Coast Region of Tanzania, and has a population size of 217,274. It has 94 registered villages and 385 hamlets. The study is operated within the Rufiji Health and Demographic Surveillance Site (RHDSS) where about 20% of households have at least one child under the age of 3 years [18]. The prevalence of stunting and anemia among children under 5 years in Rufiji is 35 and 61% respectively [19]. Its demographic characteristics are representative of rural Tanzania, most adults are subsistence farmers who cultivate staples like maize, rice, cassava, and millet Fig. 1.

### Community-based service delivery

This study engages existing Agriculture Extension Workers (AEWs), Livestock Extension Workers (LEWs) and Community Health Workers (CHWs) to deliver the interventions. AEWs and LEWs are employed by the government to train and support farmers in agriculture and animal husbandry and best practices for increased productivity. CHWs are an existing cadre in many rural settings of Tanzania, but have no formal wages. The project therefore takes advantage of existing cadres of workers who are familiar with their communities.

Typically, each AEW, LEW and CHW works in one village with approximately 400 households. They conduct regular home visits among all households to which they have been assigned providing basic health education, promoting preventive health care services, and supporting best practices in agriculture and livestock production. All three forms of home-based services; health, agriculture and livestock exist in all Rufiji villages.

### Design

**Fig. 2:**
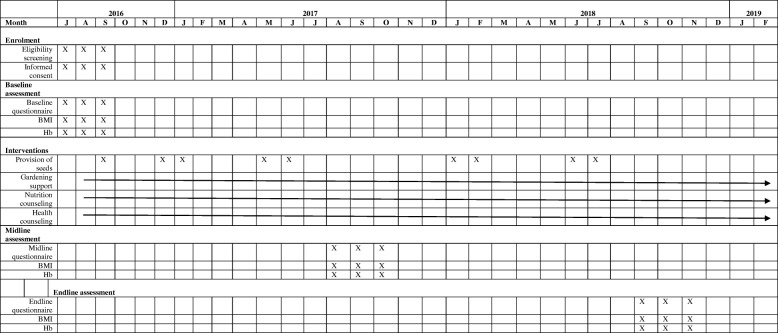
Study activities over study period

This is a 36-month long community-based prospective study carried out in 10 villages. A cluster-randomized design is used to evaluate the effects of the intervention. Data collection take place at three time points, at baseline, midline, and endline. Study activities are summarized in Fig. 2*.*

Eligibility criteria for households participating in the study are: (i) having a woman of reproductive age (18–49 years) and at least one child 6–36 months of age, (ii) having access to a plot of land or containers where vegetables could be grown, and (iii) provided informed consent.

### Randomization and sampling

The unit of randomization is a village. Ten villages within the Rufiji HDSS were randomly selected and matched based on location, proximity to water, and population size. One village in each matched-pair was randomly assigned to receive the intervention (five villages) and the other to the control group (five villages).

The Rufiji HDSS dataset was used in random sampling of households with at least one woman of reproductive age and at least one child 6–36 months of age in all study villages. We considered a proportional-based representation of households based village size in the random selection of households. Thus, a total of 1006 households were enrolled with a ratio of 1:1 in intervention and control villages (Table 1). Enrolment was initiated on July 2016 and the endline survey is planned for July 2019 Fig. 2.

**Table 1:** Sample frame of randomized study villages and households

Intervention villages	Village population size	Number of household with 6–36 months child	Total enrolment
Kibiti A	837	286	170
Mchukwi B	455	129	64
Mgomba North	386	194	134
Umwe North	558	184	71
Umwe South	298	97	65
Total	2534	890	504
Control villages			
Kibiti B	1732	346	206
Mchukwi A	383	86	68
Mgomba South	326	124	104
Ikwiriri South	594	149	84
Ikwiriri Central	230	78	40
Total	3265	783	502

### Sample size

The sample size was pre-determined by the size of HDSS and the logically feasible time frame of 2 years. The number of eligible households which could be enrolled was estimated before (as 1000) to be sufficient for pilot implementation of integrated nutrition-sensitive interventions but no formal sample size calculation was performed.

### Enrolment procedure

Participating households in each study arm were screened, consented, and enrolled. Research assistants (RA) visited and screened sampled households for each study village. RAs introduced the study to the household member and verified participant’s inclusion criteria. For each eligible household, one woman and one child were enrolled in the study after providing informed consent. In case the household has more than one eligible member, simple random sampling technique (lottery method) within each household was used to select one woman. The same technique was used to select one child 6–36 months of age. RAs then proceeded with administration of electronic baseline data collection.

### Description of the interventions

Households in the intervention arm receive: (1) interventions to promote homestead food production and increase agricultural production and food diversity, (2) nutritional counselling, including prevention and management of child malnutrition with a focus on the first 1000 days, locally adapted instructions on the mix and quantity of food suitable for children ages 6–36 months, (3) a health-focused intervention, including information on micronutrient supplementation, integrated management of child illnesses, and safe water, sanitation and hygiene practices (WASH).

Households enrolled in the intervention also receive agricultural inputs such as seeds, fertilizer, and watering cans. Local Agricultural Officers identified six varieties of crops (Africa eggplant, amaranthus, spinach, tomato, okra, and Chinese cabbage) for the study and recommended specific varieties for each intervention village based on growing conditions and village preference. Each participating household in intervention villages receive three varieties of seeds at least three times during the study period.

The behavior change component of the intervention includes information on cultivation of nutrient-rich crop varieties, best practices for home gardening, safe water, sanitation, and hygiene practices (WASH), breastfeeding and complementary feeding, dietary intake and nutrition, and other basic public health messages. AEWs, LEWs and CHWs are trained on topics related to cultivation of nutrient-rich crop varieties and provision of basic public health services to households. This includes encouraging and monitoring the consumption of iron and folate supplements during pregnancy; use of zinc oral rehydration solution for management of diarrhea; promotion of hygienic and sanitary practices at the household level, promotion of preventive health behaviors such as immunization for children; and referral of sick and malnourished children to local health centers for appropriate care.

There are two delivery mechanisms for the intervention: farmer field schools and households visits. Household visits are conducted approximately every 2 weeks. Households receive separate visits from an AEW/LEW or CHW on a rotating schedule. Field schools are also held approximately every 2 weeks and attended by all three types of extension workers.

### Farmer field schools

In collaboration with village and hamlet leaders, AEWs identified one participating household farm in each of four to five hamlets per village to be used as Farmer Field Schools (FFS). Participants within a given hamlet were invited to partake in FFSs. Farmer field schools are open to the public as they also existed in these communities before the intervention took place. Households participating in the intervention are highly encouraged to attend by extension workers. The number of participants attending a FFS typically ranges from 10 to 15, determined by the number of enrolled households per hamlet. Collaboration with village and hamlet leaders ensure that the selected individuals are valued and trusted community members interested in leading fellow farmers. The FFS is designed to (i) illustrate the benefits of innovative agricultural practices to the community, (ii) improve community knowledge about nutritious crops available in the district, (iii) reinforce messages provided by during household visits, (iv) offer a forum for discussion and collaboration between farmers, and (v) empower women with knowledge and skills for increased decision-making at the household level. Model farmers use the demonstration farm as a classroom and hold organized “classes” with other women farmers in their hamlet to illustrate how to become a more productive farmer. The design of FFS enables AEWs to maintain close connections with model farmers, providing them with information about new agricultural technologies and practices as they become available in the district.

### Household visits

AEWs, LEWs and CHWs implement a system of household visits with all enrolled smallholder farming households in the community. All cadres receive basic nutritional, agricultural and maternal and child health training to ensure messages act to reinforce and complement each other. The transmission of the same messages through multiple channels aims to increase their credibility and the probability of longer term adoption of recommended behavioral change. One-on-one meetings with AEWs, LEWs and CHWs through home visit are held biweekly for the purposes of providing maximum amount of information in a feasible manner. Household visits and FFSs are staggered so that farmers receiving the intervention are continually exposed to information through one of these channels.

### Supervision

Extension workers are continuously monitored by the study field manager to sure that the above two main approaches are delivered routinely to participants as outlined in an intervention manual developed by the research team in collaboration with local officials. A list of randomly selected participating households in each intervention village is generated every week and visited by the field manager to conduct monitoring and evaluation of the intervention.

### Data collection

Data are collected by trained interviewers using surveys administered with electronic tablets at three time points, once at baseline, once 12 months after intervention implementation, and once approximately 36 months after intervention implementation. Agricultural practices and primary outcomes are also measured in a random sample of non-participant households in the intervention villages which may assist to inform spillover effect or spread of the intervention. Anthropometric measurements of women and young children take place at three time points. Hemoglobin for women and young children is measured among a subset of the study population at these time points.

### Questionnaires

Questionnaires developed by the research team include socio-demographic and economic data, self-reported recent history of illness, infant and young child feeding, dietary intake, physical activity, agriculture production, food security, and women’s empowerment. Agriculture production data include the area of specified crop production, production quantities, sales of produce and income realized, purchase and use of agriculture inputs, and other operational expenses. Dietary intake is assessed using a food frequency questionnaire.

### Qualitative data collection

Qualitative methods are used to assess the reach of the intervention, opportunities and challenges. Focus group discussions will be conducted with study participants and non-study participants in intervention villages. Key informant interviews will also be conducted with health care providers, extension workers, and village and district officials. Interviews and focus groups aim to ascertain participants’ experiences with the intervention and suggestions for improvement in the delivery and sustainability of the intervention. In addition, the interviews and discussion will also identify bottlenecks and sources of improvement or adaption of homestead agriculture and nutrition interventions in the community.

### Outcomes

The primary outcome for the study is women’s and children’s dietary diversity (number of food groups consumed out of 10) [20, 21]. Secondary outcomes include women’s and children’s anemia status (hemoglobin [Hb] levels < 12 g/dl for non-pregnant women and Hb < 11 g/dl for pregnant women, and Hb < 11 g/dl for children); child growth (weight for age z-score [WAZ], weight for height z-score [WHZ], height for age z-score [HAZ], and mid upper arm circumference [MUAC]); women’s growth (body mass index, and MUAC); early childhood development (Caregiver Reported Early Development Index [CREDI]) [22]; and reach and extent of intervention-promoted practices and behaviors (home gardening and food consumption).

### Data analytical plan

Data will be analyzed based on an intent-to-treat strategy. Data will be classified into three main categories; (i) the primary outcome variables including women and children dietary diversity, (ii) secondary outcomes including women’s and children’s growth and anemia status; and (iii) other covariates including socio-demographic characteristics, village, age, gender, health status, WASH practices, and crop production and practices, and livestock ownership. In addition, standard tools to assess food security (food reserve availability index), dietary diversity in women of reproductive age and children, women empowerment in agriculture index, water and sanitation (WASH) practices as well as household asset index will be incorporated, and aggregate data used to predict vulnerable households.

Height (or length) and weight will be standardized based on 2006 WHO child growth standards to create HAZ, WAZ and WHZ scores. Biological implausible values based on WHO recommended cutoffs at 6 SD will be eliminated.

Generalized linear mixed models with random intercepts and robust standard errors will be used to assess for continuous repeated primary outcomes. Generalized linear mixed models with random intercepts and robust standard errors will be used to assess repeated primary outcomes.

### Study status

Enrolment into the study began in July 2016 and completed in November 2017. The intervention period is August 2016 through July 2019. The study is ongoing.

## Discussion

Multi-sectoral cadres of extension workers may be effective in addressing malnutrition among rural women and children in Tanzania. Most LMICs face severe health workforce shortages, hampering the implementation of nutrition and health initiatives and attainment of health goals [23]. Many rural communities seek health care from unlicensed traditional healers and traditional birth attendants [24], highlighting an urgent need for innovative delivery approaches. Recent reviews support integration of nutrition interventions into agricultural extension and advocacy services for maximum nutrition impact in the community [17]. Engagement of non-health sector workers, particularly agricultural extension workers, together with community health workers, may help to alleviate nutrition and health challenges in the community.

This study will contribute to a growing body of evidence on a knowledge gap that is central to maternal and child health implementation research: how agriculture programs can be designed and harnessed to improve nutrition. This study will test the effectiveness of an integrated nutrition-sensitive intervention with a focus on homestead food production for enhancing women’s empowerment, increasing income, improving crop and dietary diversity, and ultimately improving nutritional and health outcomes. This evaluation will demonstrate the feasibility and potential of nutrition-sensitive approaches for improving nutrition and health among vulnerable populations in rural Tanzania.

There is a possibility of bias whereby study villages may have different nutrition indicators as well as varied climatic conditions for crop production, including potential difference in access to water. We have minimized this bias by pairing control and intervention villages based on proximity to water sources. Issues of sustainability include the feasibility of this workload on extension workers after the study period has concluded. Currently, this study is providing logistical and financial support to participating workers to supplement the extra workload. Agriculture extension workers are paid by the district and therefore could continue these visits as part of their normal workload, but CHWs are typically volunteers. Our study will examine the effectiveness of the proposed intervention as a proof-of-principal and we will use the experience and findings generated from this study to engage with policy makers in Tanzania about next steps, including the possibility of refining the intervention and further scale up with additional evaluation of effectiveness and cost-effectiveness in the next phase of this effort.

## Abbreviations

AEWs: Agriculture extension workers
BMI: Body mass index
CHWs: Community health workers
CREDI: Caregiver reported early development index
FFS: Farmer field schools
HAZ: Height for age z-score
HB: Hemoglobin
HDSS: Health and demographic surveillance site
HFP: Homestead food production
LEWs: Livestock extension workers
LMICs: Low and middle-income countries
MUAC: Mid upper arm circumference
RAs: Research assistants
WASH: Safe water, sanitation and hygiene practices
WHO: World Health Organization
WHZ: Weight for height z-score
